# Identification of Crucial Modules and Genes Associated with Bt Gene Expression in Cotton

**DOI:** 10.3390/genes15040515

**Published:** 2024-04-19

**Authors:** Guiyuan Zhao, Zhao Geng, Jianguang Liu, Haiyan Tian, Xu Liu, Zetong An, Ning Zhao, Hanshuang Zhang, Liqiang Wu, Xingfen Wang, Yongqiang Wang, Guiyin Zhang

**Affiliations:** 1 State Key Laboratory of North China Crop Improvement and Regulation, Key Laboratory for Crop Germplasm Resources of Hebei, College of Agronomy, Hebei Agricultural University, Baoding 071001, China; zhaogy0302@163.com (G.Z.); wuliqiangwu@126.com (L.W.); cotton@hebau.edu.cn (X.W.); 2 Key Laboratory of Cotton Biology and Genetic Breeding in Huanghuaihai Semiarid Area, Ministry of Agriculture and Rural Affairs, Institute of Cotton, Hebei Academy of Agriculture and Forestry Sciences, Shijiazhuang 050051, China; gengzhao1006@163.com (Z.G.); liujianguangbj@126.com (J.L.); tianhaiyan8010@163.com (H.T.); 17734567226@163.com (X.L.); 15617133929@163.com (N.Z.); hanshuangzhang@126.com (H.Z.); wangyongqiang502@126.com (Y.W.)

**Keywords:** cotton, molecular breeding, *Bacillus thuringiensis* toxins, WGCNA

## Abstract

The expression of *Bacillus thuringiensis* (Bt) toxins in transgenic cotton confers resistance to insect pests. However, it has been demonstrated that its effectiveness varies among cotton cultivars and different tissues. In this study, we evaluated the expression of Bt protein in 28 cotton cultivars and selected 7 cultivars that differed in Bt protein expression for transcriptome analysis. Based on their Bt protein expression levels, the selected cultivars were categorized into three groups: H (high Bt protein expression), M (moderate expression), and L (low expression). In total, 342, 318, and 965 differentially expressed genes were detected in the H vs. L, M vs. L, and H vs. M comparison groups, respectively. And three modules significantly associated with Bt protein expression were identified by weighted gene co-expression network analysis. Three hub genes were selected to verify their relationships with Bt protein expression using virus-induced gene silencing (VIGS). Silencing *GhM_D11G1176*, encoding an MYC transcription factor, was confirmed to significantly decrease the expression of Bt protein. The present findings contribute to an improved understanding of the mechanisms that influence Bt protein expression in transgenic cotton.

## 1. Introduction

Transgenic cotton expressing an insecticidal protein derived from *Bacillus thuringiensis* (Bt) is among the most rapidly adopted genetically modified crops worldwide [1,2]. Over the past three decades, transgenic Bt cotton has proved to be effective in controlling lepidopteran pests and highly beneficial to the grower and the environment by reducing the use of chemical insecticide sprays and preserving populations of beneficial arthropods [2]. 

The efficacy of Bt cotton against insect pests is dependent on the expression of Cry genes and the synthesis of the insecticidal protein [3]. However, the Bt toxin content varies among Bt-transgenic cotton cultivars, resulting in differences in the efficacy of insect resistance [4]. In addition, the efficacy of Bt-transgenic cotton against lepidopteran pests is inconsistent over the course of the growing season [5]. The main factors that lead to variable expression of a Bt transgene are its base sequence, the number of transgene copies, the promoter used, and the insertion point of the promoter and gene [6]. Xia et al., (2005) observed that a low expression level of a Bt insecticidal gene in advanced developmental stages is associated with changes in the methylation status of the 35S promoter region [7]. The Bt toxin concentration is strongly associated with nitrogen and carbon metabolism. Bruns and Abel (2003) reported a significant correlation between the Bt toxin concentration and the whole-plant nitrogen concentration in Bt-transgenic maize [8]. In Bt-transgenic cotton, production of the toxin protein is influenced by the interaction of CO_2_ and nitrogen. Moreover, elevated CO_2_ concentrations lead to a decrease in nitrogen allocation to the Bt toxin [9].

Most transgenic pest-resistant cotton cultivars have been bred by conventional crossing, which has resulted in the derivation of the majority of Bt cotton cultivars from a single transformation event. However, the cotton cultivars in production differ in the efficacy of their insect resistance. Adamczyk et al., (2004) investigated the differences in Bt transgene expression among Bollgard cotton cultivars and observed that the genetic background has a significant impact on Cry1Ac expression [10]. Cheema et al., (2016) evaluated the genotypes and protein expression levels of 52 Bt cotton accessions and determined that among the genotypes derived from the same type of transformation event, the toxin concentrations differed significantly; the authors speculated that the genetic background plays an important role in transgene expression [11]. Understanding the molecular mechanisms that regulate Bt transgene expression will assist in breeding cotton cultivars that exhibit high Bt protein expression.

Weighted gene co-expression network analysis (WGCNA) is a statistical method used to analyze the intricate interactions between genes and phenotypes [12]. This approach is based on gene correlations and has been employed to construct gene networks, identify modules/subnetworks, and detect hub genes within modules. This procedure has been proven to be an efficient data-mining method in various plant species [13,14,15]. In the present study, we investigated the Bt protein contents of 28 transgenic cotton cultivars harboring a single Bt gene, Cry1Ac, and analyzed the transcriptomes of 7 selected cultivars. By conducting a WGCNA, we aimed to identify gene co-expression network modules and hub genes associated with Bt transgene expression.

## 2. Materials and Methods

### 2.1. Materials and Bt Protein Content Analysis

The seeds of 28 Bt cotton (*Gossypium hirsutum* L.) cultivars carrying a single Bt gene, Cry1Ac, driven by the Cauliflower mosaic virus 35S promoter were collected from our laboratory. All cultivars had been self-pollinated for at least six generations. Leaves were sampled from the fourth node from the bottom 60 days post-sowing for the measurement of the Bt protein content and RNA sequencing. To quantify the toxin, the leaf samples were placed in an Eppendorf tube in 500 µL of 1× extraction buffer (EnviroLogix, Inc., Portland, ME, USA) and ground manually. The homogenate was centrifuged at 12,000 rpm for 5 min, and the supernatant was retained for the following procedure. An enzyme-linked immunosorbent assay (ELISA) was performed using a QualiPlate kit for Cry1Ab/Cry1Ac (EnviroLogix) following the manufacturer’s protocol. Quantification of the toxin protein was conducted by generating a standard curve of the absorbance values of the test samples and a standard (purified toxin protein) using simple regression analysis. The protein content calculation formula was Cry1Ab/Cry1Ac protein content (ng/g fresh weight) = test sample concentration (ppb) × dilution factor/tissue fresh weight (g). Three biological replicates and three technical replicates were performed for each biological sample.

### 2.2. RNA Sequencing Data Generation and Processing

Based on the Bt protein contents, we selected seven cultivars for RNA sequencing (RNA-seq). For each cultivar, three independent biological replicates were collected, resulting in 21 samples in total. Total RNA was extracted from the sampled leaves and sent to Wuhan SeqHealth Technology Co., Ltd. (Wuhan, China). Paired-end sequencing was performed using an MGISEQ-T7 sequencer. The raw transcriptome sequence data were uploaded into an NGDC repository with accession number PRJCA024190 (https://ngdc.cncb.ac.cn/bioproject/browse/PRJCA024190, accessed on 10 March 2024). Quality control for the sequencing data was performed with FASTQC version 0.23.0. Clean reads were aligned to the cotton reference genome Gossypium_hirsutum_NDM8HEBAU (https://www.cottongen.org/, accessed on 9 August 2021) with HISAT2 version 2.1.0 [16,17]. Genes differentially expressed between different groups were detected using DESeq2 v 1.24.0 software [18]. Genes with a log2 fold-change ≥ 1 and a false discovery rate ≤ 0.05 were considered to be significantly differentially expressed genes (DEGs). Gene ontology (GO) and Kyoto Encyclopedia of Genes and Genomes (KEGG) pathway enrichment analyses of the DEGs were performed using clusterprofiler (significant enrichment was based on a corrected *p*-value < 0.05) [19].

### 2.3. Weighted Co-Expression Network Construction

Co-expression analysis was performed using the WGCNA package for R3.2 [12]. The top 50% most variable genes, as determined by a variance analysis, were screened to construct a co-expression network. The network topology analysis ensured a scale-free topology network with a defined soft-thresholding power of 6. Using the dynamic tree cutting algorithm, we identified the total number of modules with the parameters minModuleSize = 200 and mergeCutHeight = 0.25. The correlation coefficients between the eigengene values of the modules and the Bt protein contents were calculated to identify the modules that were strongly associated with the Bt protein. The network of critical selected genes was visualized using Cytoscape version 3.9.1 [20]. The genes with the highest kME (characteristic gene connectivity) values were selected as the critical genes in each module. 

### 2.4. Virus-Induced Gene Silencing of Hub Genes

For the virus-induced gene silencing (VIGS) assay, part of the coding sequence (300 bp fragments) of hub genes was amplified by PCR using Ji172 cotton cDNA. The fragment was inserted into a Tobacco rattle virus2 (TRV2) vector to construct a TRV–hub gene recombinant plasmid by infusion technology (General Biology Co., Ltd., Anhui, China), which was then transformed into Agrobacterium tumefaciens strain GV3101. The transformed GV3101 strains carrying the TRV–hub gene vector, TRV:*GhCLA1* (indicator vector), or TRV:00 (empty vector) were mixed with a strain harboring the pYL192 plasmid (helper vector) (1:1 ratio, OD600 = 0.8) and co-injected into two fully expanded cotyledons of cotton seedlings through their wounding sites using a 1 mL needleless syringe. The infiltrated plants were cultivated at room temperature under dim light conditions overnight. The plants were then grown in a growth chamber under a 16 h light/8 h dark cycle at 23 °C. When the true leaves of plants silenced by TRV:*GhCLA1* started to display the albino phenotype, quantitative reverse-transcription PCR (RT-PCR) was used to detect the silencing efficiency. After about 12 days (when the leaves of the positive control plants (TRV:*GhCLA1*-transfected plants) showed an albino phenotype), leaves from the TRV–hub gene- and TRV:00-transfected seedlings were randomly selected to quantify their expression levels of the hub gene by qRT-PCR, and the Bt protein contents were quantified by ELISA according to the above method. The VIGS assay was conducted with three biological replicates. 

### 2.5. Quantitative Reverse-Transcription PCR

Total RNA was isolated from each sample as described above. The first-strand cDNA synthesis of mRNA was performed using a Fastking RT kit, and qRT-PCR was carried out using SuperReal PreMix Plus (Tiangen, Beijing, China). Histone was used as an internal reference gene for internal normalization. The reaction conditions were set as follows: 95 °C for 10 min, followed by 40 cycles of 95 °C for 15 s and 60 °C for 1 min, followed by a final dissociation curve analysis. All reactions were performed with three biological replicates per sample. The relative expressions were calculated using the comparative CT (2^−ΔΔCt^) method. The primers used are listed in Table S1. The protein of Cry1Ac was extracted according to the above method.

### 2.6. Statistical Analysis

The experiments were performed in triplicate, and the results are reported as mean values, with error bars showing means ± SDs. Statistical analysis of the experimental data was performed using Microsoft Excel version 2013. The *t*-test method was employed to assess the differences between the investigated variables. The significance level was set as ** *p* < 0.01 and * *p* < 0.05 compared to control.

## 3. Results

### 3.1. Quantification of Cry1Ac Bt Toxin

**Figure 1:**
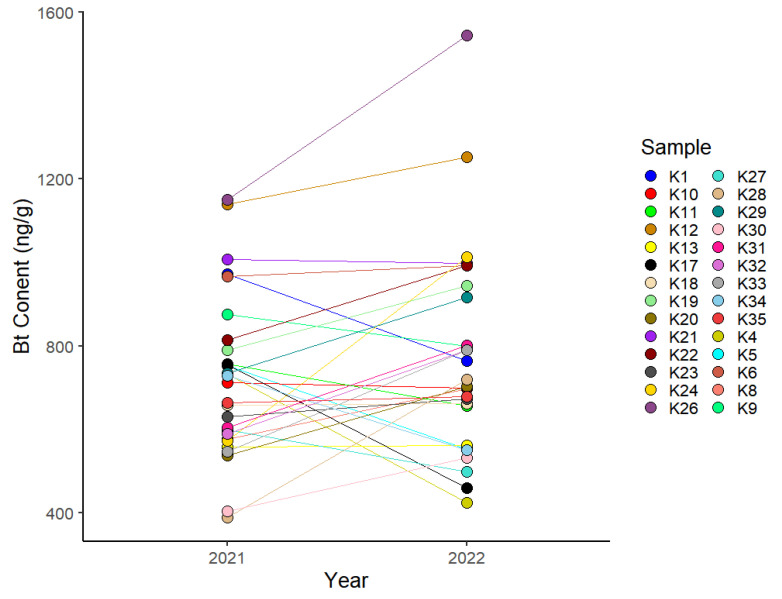
Bt protein contents of 28 cultivars in 2021 and 2022.

The Bt protein contents of 28 cultivars of cotton were analyzed in 2021 and 2022. The overall correlation coefficient for the Bt contents of the cultivars between the two years was 0.64, and the Bt contents of different varieties varied from 0.46 to 84.2 between the two years. The Bt protein contents of certain cultivars (e.g., K13, K18, and K21) were relatively stable between the two years, whereas those of other cultivars differed substantially between the two years; for example, the Bt content of K28 was 390.5 ng/g in 2021 and 719.4 ng/g in 2022. We noted that the Bt protein contents of K12, K21, and K26 were relatively high in both years, exceeding 1000 ng/g, except for that of K21, which was slightly less than 1000 ng/g in 2021. In contrast, the Bt protein contents of K13 and K30 were relatively low (less than 600 ng/g) in both years Figure 1 and Figure S1 and Table S1). Based on the Bt protein content results, seven cultivars that showed relatively stable Bt protein expression, comprising K10, K12, K13, K21, K23, K26, and K30, were selected for subsequent transcriptome analysis.

### 3.2. RNA-Seq Data and Analysis

To explore the molecular basis of differences in insect resistance among Bt-transgenic cotton cultivars, RNA-seq analyses were conducted. The seven cultivars selected according to their Bt protein contents were divided into three groups: the H group (high Bt protein contents; K12, K21, and K26), M group (moderate Bt protein contents; K10 and K23), and L group (low Bt protein contents; K13 and K30). After removing low-quality data, 38,225,860–56,284,598 clean reads were retained for each library, with a Q30 exceeding 95.76%. Of these reads, 97.97–99.17% were mapped to the *G. hirsutum* reference genome sequence. In total, 342 (150 upregulated and 192 downregulated), 318 (149 upregulated and 169 downregulated), and 965 (434 upregulated and 531 downregulated) DEGs were detected in the L vs. H, L vs. M, and M vs. H comparison groups, respectively (Table S2). Only one gene, encoding a glycine-rich protein, *GhM_A07G2431*, was common to all three comparison groups. We noticed that *GhM_A07G2431* gene expression was significantly higher in the H group cultivars, with high expression of Bt, than in the L group, with low expression of Bt protein, suggesting that it may play an important role that affects the Bt protein content.

**Figure 2:**
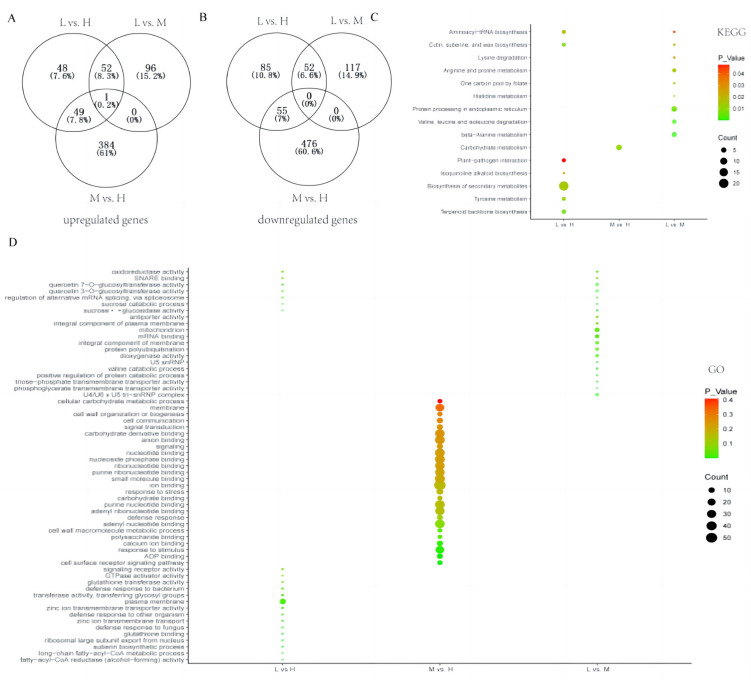
RNA-seq analysis of comparisons between different Bt contents of varieties (L vs. H, L vs. M, and M vs. H). (**A**) A Venn diagram showing upregulated DEGs of different comparisons. (**B**) A Venn diagram showing downregulated DEGs of different comparisons. (**C**,**D**) KEGG and GO enrichment analyses of DEGs of different comparisons.

The DEGs were functionally annotated on the basis of GO and KEGG pathway enrichment analyses (Figure 2). The KEGG pathway enrichment analysis indicated that “Aminoacyl–tRNA biosynthesis” and “Cutin, suberine, and wax biosynthesis” were significantly enriched pathways common to the L vs. H and L vs. M comparisons. The most highly enriched KEGG pathways among the DEGs in the L vs. H comparison were “Biosynthesis of secondary metabolites”, “Tyrosine metabolism”, “Terpenoid backbone biosynthesis”, “Isoquinoline alkaloid biosynthesis”, and “Plant–pathogen interaction”. The GO terms “oxidoreductase activity”, “quercetin 7-O-glucosyltransferase activity”, “SNARE binding”, “quercetin 3-O-glucosyltransferase activity”, “regulation of alternative mRNA splicing, via spliceosome”, “sucrose α-glucosidase activity”, and “antiporter activity” were common to the L vs. H and L vs. M comparisons. No GO terms were common to the other comparisons. The majority of the DEGs were amino acid metabolism-related genes, suggesting that a difference in amino acid metabolism ability was the main factor leading to the differential Bt protein contents among the cultivars. 

### 3.3. Identification of Crucial Modules

**Figure 3:**
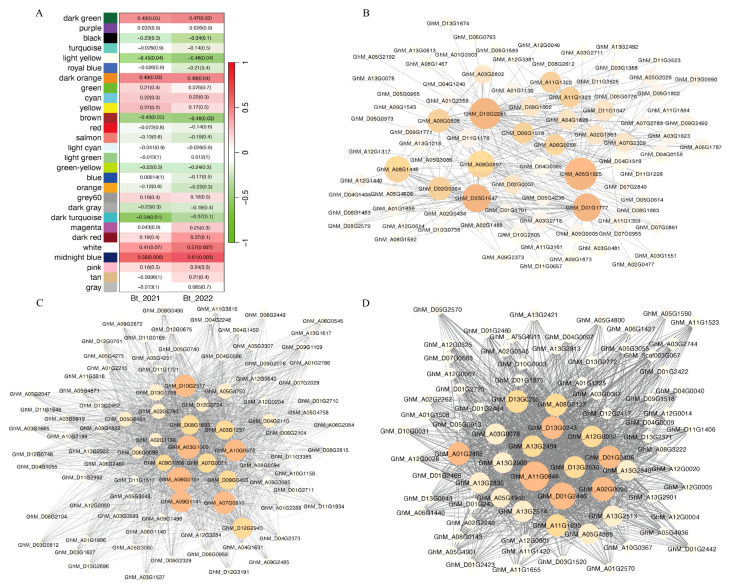
Construction and identification of co-expression modules. (**A**) Correlation heatmap of modules and Bt contents. The adjusted *p*-value and correlation coefficient are shown in each cell. (**B**) Networks of hub genes in the midnight blue module. (**C**) Networks of hub genes in the white module. (**D**) Networks of hub genes in the dark turquoise module.

To identify the hub genes associated with expression of the Bt protein, the Bt protein contents in 2021 and 2022 were used as phenotypic data for WGCNA. The WGCNA clustered genes into modules based on the correlations among their expression patterns. In total, 33,550 expressed genes were divided into 28 modules, and each module comprised 243 to 4919 genes. Among these modules, the white module and midnight blue module were positively correlated with the Bt protein content. The dark turquoise modules were negatively correlated with the Bt protein content (Figure 3A).

### 3.4. Functional Enrichment Analysis of the Genes in the Crucial Modules

We conducted an enrichment analysis for the three aforementioned modules to further explore their biological functions. Specifically, the 243 genes in the white module were significantly enriched in “Transcription machinery”, “RNA polymerase”, and “DNA repair and recombination proteins”. The 594 genes in the midnight blue module were significantly enriched in “Protein phosphatases and associated proteins”. No significantly enriched pathway was identified in the dark turquoise module.

To identify hub genes involved in Bt transgene expression, we further screened these three modules using the module membership (kME) value. We selected the 20 genes with the highest kME values as hub genes in each module. In the midnight blue module, the hub gene with the highest edge number, *GhM_D10G2281*, was annotated as the NUDIX hydrolase domain. Other highly connected hub genes, including SKP, MYC, Exostosin-like, Lipoxygenase, and Glycoside hydrolase, have been reported to play important roles in plant development and stress responses. In the white module, the top hub genes contained many enzymes related to transcription regulation, such as RNA helicase, RNA polymerase, and transferase. In the dark turquoise module, the top 20 hub genes were mainly related to dehydratase and hydrolase (Figure 3B–D and Table S3).

### 3.5. Identification of the Hub Gene Associated with Bt Protein Content

**Figure 4:**
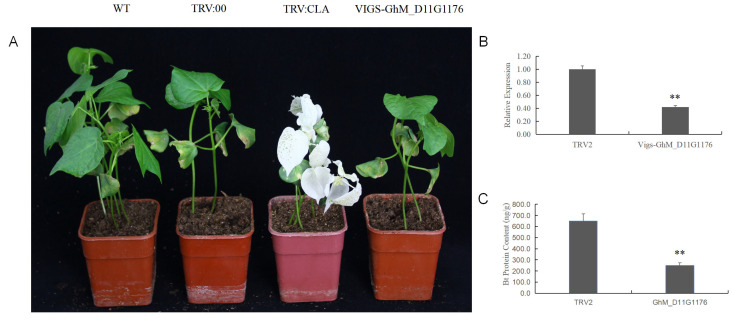
Silencing of *GhM_D11G1176* by VIGS. (**A**) Phenotypes of the VIGS-*GhM_D11G1176* and control cotton plants. (**B**) The expression of *GhM_D11G1176* was determined by qRT-PCR after the VIGS injection. (**C**) The Bt contents in control and VIGS-*GhM_D11G1176* cotton plants. Significance was determined by *t*-test (** *p* < 0.01).

To examine the influence of the hub genes on the Bt protein content, three genes, namely, *GhM_D01G2446*, *GhM_D11G1176*, and *GhM_A05G1925*, were randomly selected and the VIGS approach was used to silence their expression. The vectors TRV:GhCLA1 and TRV:00 were used as a positive control and a negative control, respectively. The degree of albinism of the positive control plants revealed the effectiveness of the VIGS system, and the gene-silencing effect was verified by qRT-PCR. The gene *GhM_D11G1176* was annotated as an MYC transcription factor. When the *GhM_D11G1176* expression level in the VIGS-*GhM_D11G1176* plants decreased significantly, the Bt protein was detected. The Bt protein content in the VIGS-*GhM_D11G1176* plants was significantly lower than that in the control TRV:00 plants (Figure 4), indicating that the expression level of *GhM_D11G1176* affected the Bt protein content. The gene *GhM_D11G2446* encoded a NUDIX hydrolase, and *GhM_A05G1925* encoded a glycoside hydrolase. Both genes were also included in the VIGS experiment, but the protein content of the gene-silenced plants was not significantly different from that of the control plants, suggesting that the expression of these two genes did not affect Bt protein expression or accumulation.

## 4. Discussion

Transgenic Bt cotton is the predominant commercial transgenic crop in China that has been cultivated for the longest period. Expression of the exogenous Bt protein can effectively improve the insect resistance of transgenic plants. However, the mechanism regulating the expression of the Bt protein is complex. The copy number, promoter type, and insertion position of the gene may affect the expression level of the protein. However, in this study, we evaluated the Bt protein contents of 28 transgenic cotton varieties from the same transformation event, excluding the influence of these factors. These materials had the same Bt gene but different genetic backgrounds. The protein content results showed significant differences in protein expression levels among different cultivars in the same environment, with the highest being over 1000 ng/g and the lowest being less than 400 ng/g, suggesting that the genetic background has an important impact on the expression of Bt. In addition, we noticed that some varieties varied greatly between different years, indicating that the environment also has a significant impact on the expression of Bt genes (Figure 1 and Table S1). Previous studies suggested that transgene expression in transgenic crops is significantly influenced by environmental factors. For example, the application of a high dose of nitrogen fertilizer increases Bt endotoxin expression by 14% compared with that under a low dose [21]. In addition, significant decreases in Bt protein abundance under salt stress [22], high temperatures [23], water deficiency, and waterlogging [24] have been reported. Further evaluation is needed to determine whether varieties with protein contents that are less affected by the environment have stronger environmental adaptability. In short, the adaptability of plants to their natural environment is influenced by their genetic backgrounds, so the selection of donor parents in cross-breeding is crucial for the development of cultivars that stably express Bt protein at high levels.

In this study, we selected seven cultivars for transcriptome analysis and divided them into three groups (high, medium, and low), according to the Bt protein contents, for comparison. A total of 342, 318, and 965 DEGs were detected in each comparison group. In previous studies, one material or two materials were usually used for comparisons before and after stress treatments. However, in general, this cannot exclude different genetic backgrounds and the resulting metabolic differences among varieties. This study involved a direct comparison of transcriptomic data among seven cultivars, enabling the exclusion of additional genetic backgrounds. Therefore, the number of differential genes was relatively low.

To explore the mechanism that controls Bt protein expression, we used WGCNA to analyze the transcriptomes of seven cultivars and identified crucial hub genes. In this study, the Bt protein content decreased significantly after the *GhM_D11G1176* gene encoding the MYC transcription factor was silenced using the VIGS approach. MYC transcription factors play a central role in the jasmonic acid (JA) signal transduction pathway. These factors are crucial for diverse processes, such as growth, development, secondary metabolite synthesis, stress responses, and phytohormone signal transduction. MYC transcription factors play a regulatory role in the JA pathway in response to wounding and pest attacks. Tomato *SlMYC2* acts downstream of the JA receptor to orchestrate JA-mediated activation of the responses to wounding and pathogen attacks [25]. Transgenic rice overexpressing *OsMYC2* shows enhanced resistance to leaf blight [26]. In addition, MYC transcription factors play an important role in plant responses to abiotic stresses. The rice MYC family gene *OsbHLH148* improves drought tolerance mediated through the JA signaling pathway [27]. Expression of *SlMYC2* is increased in response to cold stress, enhancing tolerance to low temperatures in tomato. Silencing *SlMYC2* reduces antioxidant enzyme activity and increases cold-induced injury [28]. Furthermore, Arabidopsis *AtMYC2* functions in conjunction with *AtMYC3* and *AtMYC4* to control leaf senescence [29], flowering [30], and chlorophyll degradation [31]. These results indicate that MYC transcription factors play important roles in plant resistance, growth, and adaptation to environmental changes and directly affect the Bt protein content. It is important to note that the MYC transcription factor is not the sole gene influencing the expression of Bt. Other genes associated with stress resistance, growth, and development may also play a role in regulating the expression of BT. Further research is necessary to fully understand these complex mechanisms. Therefore, improving the adaptability of Bt-transgenic cotton plants to maintain their general health and vigor may be essential to realize the full potential of transgenes.

## 5. Conclusions

In the current research, we evaluated the expression of Bt protein in 28 cotton cultivars and constructed a gene-weighted co-expression network to detect crucial gene modules and hub genes that are strongly associated with the Bt protein content. Two modules with positive correlations and three modules with negative correlations were distinguished. A candidate gene, *GhM_D11G1176*, encoding an MYC transcription factor, was confirmed by VIGS to significantly affect the expression of Bt protein. The present findings contribute to an improved understanding of the mechanisms that influence Bt protein expression in cotton.

## Data Availability

The data presented in this study are available in this article and the Supplementary Materials.

## Supplementary Materials

The following supporting information can be downloaded at https://www.mdpi.com/article/10.3390/genes15040515/s1, Table S1: Primer used in qRT-PCR validation; Table S2: Bt protein contents of 28 cultivars in 2021 and 2022; Table S3: List of DEGs in the L vs. H, L vs. M, and M vs. H comparison groups; Table S4: List of hub genes in the white, midnight blue, and dark turquoise modules; Figure S1: The variation in the Bt protein contents of 28 cotton cultivars between 2021 and 2022.
