# Supplementary figures and images for: Identification of Crucial Modules and Genes Associated with Bt Gene Expression in Cotton
Source: Genes (Basel). 2024 Apr 19;15(4):515. doi: 10.3390/genes15040515 (PMC11050609; doi:10.3390/genes15040515)

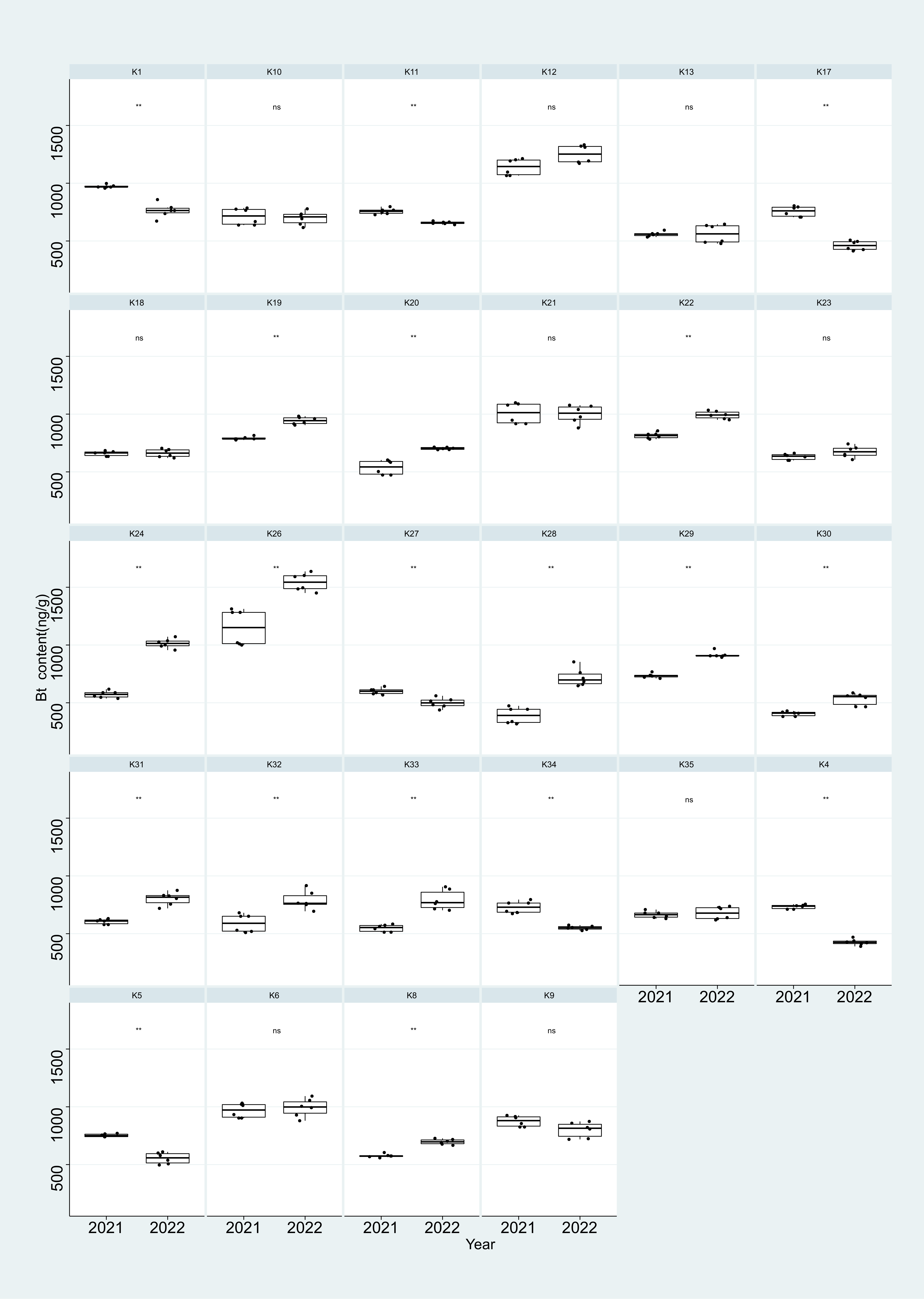

Supplement: Supplementary file 1 [file genes-15-00515-s001.zip › Figure S1.png]
